# A Literature Review of the Integration of Ancient Indian Mythology in Clinical Medicine: A Holistic Approach to Health and Healing

**DOI:** 10.7759/cureus.63779

**Published:** 2024-07-03

**Authors:** Shwetha C Pondomatti, Ishaan Tyagi, Kirtika K Shrivastava, Swati Mahajan, Jitendra Patel, Manjusha A Shinde

**Affiliations:** 1 Physiology, Subbaiah Medical College, Shivamogga, IND; 2 Internal Medicine, Dr. D. Y. Patil Medical College, Hospital & Research Centre, Pune, IND; 3 Physiology, People's College of Medical Sciences and Research Centre, Bhopal, IND; 4 Physiology, Gujarat Medical Education and Research Society Medical College, Godhra, IND; 5 Physiology, Gujarat Medical Education and Research Society Medical College, Vadnagar, IND; 6 Physiology, All India Institute of Medical Sciences, Kalyani, IND

**Keywords:** sushruta, charaka, clinical medicine, surgical techniques, personalized medicine, dhanvantari, ayurveda

## Abstract

The fusion of mythology and ancient Indian medicine, particularly Ayurveda, is a fascinating synthesis of cultural heritage and scientific endeavor. Ayurveda encompasses a wide range of practices, including pharmacology, anatomy, physiology, surgery, and obstetrics, and integrates the rich tapestry of Hindu mythology, providing a comprehensive understanding of health and disease. The inclusion of mythological figures and narratives in the discourse of ancient Indian medicine offers a unique perspective on the integration of spiritual and empirical knowledge, highlighting the role of mythology in shaping the foundational principles of clinical medicine. The discourse explores the profound impact of Ayurveda and its mythological underpinnings on contemporary clinical practices, underscoring the timeless wisdom embedded in ancient narratives. These stories represent the bedrock of holistic medical practices, emphasizing the parity between mind, body, and spirit that is increasingly validated in modern therapeutic paradigms. The philosophy and methods detailed in the age-old texts of Sushruta and Charaka, coupled with the allegorical tales of Dhanvantari and Bharadwaja, contribute significantly to the foundational principles underpinning today's holistic medical approaches. The enduring legacy of Ayurveda and its mythological narratives continues to influence and inspire a holistic approach to health care, underscoring the indelible connection between ancient wisdom and modern medical practices.

## Introduction and background

The interweaving of mythology with the practice of ancient Indian medicine represents a fascinating fusion of cultural heritage and scientific endeavor. At the heart of this synthesis lies Ayurveda, a system of medicine that has sustained its relevance and application over thousands of years [1]. Ayurveda includes different practices like pharmacology, life structures, physiology, surgery, and obstetrics, consolidating Hindu mythology for a comprehensive understanding of well-being and maladies. The inclusion of mythological figures and narratives in the discourse of ancient Indian medicine offers a unique perspective on the integration of spiritual and empirical knowledge, highlighting the role of mythology in shaping the foundational principles of clinical medicine [1,2].

As this article unfolds, it delves into the historical foundations of Ayurveda, examining how mythological figures and stories contribute to the development of medical practices. It will explore how ancient texts, such as the Charaka Samhita and Sushruta Samhita, incorporate a wealth of information on pathology and surgery, including pioneering techniques in cataract surgery, rhinoplasty, and other medical specialties [1]. Through the lens of mythology, the article will also investigate the symbolic and metaphorical dimensions of traditional medicine, examining how the concepts of doshas, dhatu, and marmas inform diagnostic and therapeutic practices. Finally, it will consider Ayurveda's enduring legacy and the contemporary relevance of integrating ancient wisdom into modern clinical practice, offering insights into the timeless nature of ancient Indian medicine [1,3-5].

## Review

Historical foundations of Ayurveda

Ayurveda, often considered the oldest healing science, has its roots deeply embedded in ancient Indian philosophy and is believed to have originated more than 5,000 years ago [6]. The foundational principles of Ayurveda were laid down by the philosophical schools of Vaisheshika and Nyaya, alongside the Samkhya framework, during the 2nd century BC (before Christ) [7]. These schools significantly shaped the diagnostic and treatment processes in Ayurveda, emphasizing a logical and empirical approach to medicine.

Origins and Ancient Texts

The divine origin of Ayurveda is attributed to the Hindu God, Brahma, who is referred to as the creator of the universe. It is said that Brahma passed this holistic knowledge to the sages, who then disseminated it to humanity through oral teachings and writings. This transmission of knowledge was eventually documented in the Vedas, the ancient Indian scriptures, which include the Rig Veda, Yajur Veda, Sam Veda, and Atharva Veda. The Rig Veda, among these, lists numerous medicinal plants and their uses, highlighting the extensive herbal knowledge that formed a part of Ayurvedic medicine.

Significant texts such as the Charaka Samhita and Sushruta Samhita were later compiled; these texts not only encompass a wide range of medical knowledge from diagnostics to surgery but also reflect the integration of Ayurvedic principles with philosophical insights. The Charaka Samhita, for instance, is a comprehensive text on internal medicine, while the Sushruta Samhita is renowned for its detailed descriptions of surgical techniques and instruments [7].

Philosophical Underpinnings

The philosophical underpinnings of Ayurveda are largely derived from the Sāṃkhya and Nyāya-Vaiśeṣika schools of thought, which articulate a detailed classification of the physical and metaphysical world. These schools introduced concepts such as the prakriti (primordial nature) and the three guṇas (qualities) of sattva, rajas, and tamas, which are essential for understanding human constitution and psychopathology in Ayurveda.

Furthermore, the integration of the Panchamahabhuta theory (five elements theory), which includes the five elements - space, air, fire, water, and earth - into Ayurvedic practice illustrates the deep philosophical nature of this medical system. This theory is crucial for understanding the functional principles of body and mind as it relates to health and disease.

In conclusion, the historical foundations of Ayurveda are not only fascinating from a medical perspective but also offer a rich tapestry of cultural and philosophical knowledge that has influenced the development of health sciences in profound ways. The ancient texts and philosophical schools have provided a framework that continues to inform modern Ayurvedic practices and ensures its relevance in contemporary medical discourse.

Mythological figures and stories

In the annals of ancient Indian medicine, two mythological figures stand prominently: Dhanvantari and Bharadwaja. These individuals are not merely characters in lore but pivotal to the inception and dissemination of Ayurvedic knowledge.

Dhanvantari and the Emergence of Ayurvedic Knowledge

Lord Dhanvantari, revered as the physician of the gods in both the Vedas and Puranas, holds a distinguished place in the history of Ayurveda [5]. His emergence from the churning of the cosmic ocean, bearing a bowl of nectar, symbolizes the birth of Ayurvedic medicine itself. This divine incarnation is credited with organizing the vast expanse of Ayurvedic wisdom, previously composed by Brahma, into eight accessible divisions, laying the foundation for Ayurvedic practice. Dhanvantari's teachings, which were imparted orally to his disciples, form the cornerstone of Ayurvedic medicine, emphasizing the importance of perception, authoritative scripture, inference, and analogy in the healing arts [8]. Moreover, his role as an avatar of Vishnu underscores the divine origin and sanctity of Ayurvedic knowledge, further enriching its historical and cultural significance.

Bharadwaja and the Divine Transmission of Medicine

Parallel to Dhanvantari's contributions, Sage Bharadwaja's journey to the heavens to acquire Ayurvedic knowledge from Lord Indra epitomizes the divine transmission of medical wisdom to humankind. His total submission and praise of Indra earned him the gift of Ayurveda and marked a pivotal moment in the human understanding of health and longevity. Upon his return to Earth, Bharadwaja's dedication to teaching Ayurveda exactly as he had learned it from Indra ensured the purity and continuity of this divine science among mortals. His efforts in disseminating Ayurvedic knowledge to his disciples laid the groundwork for its practice and established him as a key figure in its earthly tradition [7].

The narratives of Dhanvantari and Bharadwaja, rich with mythological significance, illuminate the divine origins of Ayurveda and its early dissemination among the sages of ancient India. These stories not only provide insight into the mythological underpinnings of Ayurvedic medicine but also highlight the enduring legacy of these figures in the continuous evolution of Ayurvedic practice [5]. Their contributions, deeply embedded in the spiritual and philosophical traditions of Hinduism, reflect the intricate blend of myth, religion, and medicine that characterizes the ancient Indian approach to health and healing.

Medical practices in mythology

Sushruta and Ancient Surgery Methods

Sushruta, recognized as the father of surgery, made groundbreaking contributions to the field of surgery through his work compiled in the Sushruta Samhita. This ancient text is celebrated for its detailed surgical knowledge, which remains relevant even today. Sushruta's surgical science, known as Shalya Tantra, covered comprehensive processes aiming at the removal of objects causing pain or misery to the body or mind. He meticulously documented surgical procedures under eight categories, including excision, scarification, and suturing, emphasizing the principles of planning, precision, and perfection.

Remarkably, Sushruta introduced reconstructive procedures for various defects and described over 300 surgical procedures, highlighting his profound understanding of surgical practices. His approach to health was holistic, considering both physical and mental well-being, achieved through balanced humor, good nutrition, and a contented state of body and mind [9]. Sushruta's innovative use of anesthesia, involving intoxicants like wine and cannabis, underscored his commitment to pain management during surgery. His contributions to plastic surgery, particularly rhinoplasty, known as the Indian flap, established him as a pioneer in the field. In Indian mythology, Lord Ganesha is a Hindu God who was killed by his father Lord Shiva in a misunderstanding and to revive him, his head was replaced by the head of an elephant, marking the first citation to transplantation and plastic surgery.

Furthermore, Sushruta's emphasis on the importance of understanding human anatomy through cadaver dissection set a precedent for medical education, encouraging systematic examination and practice of models resembling diseased body parts [9]. This ancient surgeon's work on fractures, dislocations, and postoperative physiotherapy continues to inspire orthopedic surgeons worldwide.

Charaka and Holistic Healing Approaches

Charaka, another luminary in ancient Indian medicine, edited the Charaka Samhita, a foundational text of Ayurveda that covers a wide range of medical knowledge [10]. Charaka's holistic approach to medicine emphasized understanding the body as a whole and maintaining balance among the three doshas: vata, pitta, and kapha. He advocated for treatments aimed at restoring this balance through dietary changes, herbal remedies, lifestyle modifications, and therapies like massage and detoxification.

Charaka's contributions to Ayurveda extended beyond medical practices to include scientific principles. He underscored the importance of observation, experimentation, and logical reasoning in medicine, classifying diseases based on etiology and symptoms and employing diagnostic methods such as pulse and urine examination. His understanding of the body's functions, including the classification of the total number of bones and the concept of the heart as a controlling center connected to the entire body through channels, demonstrated his advanced knowledge of anatomy and physiology [3,10].

The Charaka Samhita, refined and annotated from the original text by Agnivesha, was divided into eight parts, each containing multiple chapters that delve into various aspects of medicine, including diagnosis, fetal development, and the classification of diseases. Charaka's work not only enriched the field of Ayurveda but also laid the groundwork for a comprehensive system of health care that addressed both preventive and curative aspects [1,10,11].

Both Sushruta and Charaka's contributions to ancient Indian medicine underscore the integration of mythological wisdom with scientific inquiry, laying the foundation for a holistic approach to health and healing that continues to influence modern medical practices. Their work, deeply rooted in the philosophical and spiritual traditions of Hinduism, highlights the enduring legacy of ancient Indian medicine in shaping clinical medicine today [1,11].

Mythological tales and modern medical insights

The interplay between mythological narratives and modern medical insights is vividly illustrated in ancient Indian texts, which often encode complex health concepts within stories of gods, demons, and supernatural beings. One such story is that of Karkati, a demoness in the ancient Sanskrit text Laghu-Yoga-Vasistha. Karkati's transformation into a disease-causing entity reflects the intricate understanding of pathology and the human body's response to environmental and dietary influences in ancient Indian philosophy [12]. Karkati, originally a fearsome demoness, undergoes intense penance and is granted a boon by Brahma to become a disease, specifically targeting those who indulge in unhealthy lifestyles. This transformation from a physical being to a disease symbolizes the ancient belief in the impact of moral and physical actions on health. The narrative details how Karkati, now the disease Viṣūcikā, enters the body through the air (Prāṇa Vāyu) and affects individuals who engage in unwholesome eating and living practices [13]. This mythological tale is not merely a story but a representation of the early understanding of disease transmission and pathology. It highlights how ancient practitioners might have conceptualized diseases as entities that could invade the body, reflecting a surprisingly sophisticated grasp of what modern medicine would describe in terms of pathogens and lifestyle-related illnesses [14,15]. Another intriguing connection between mythology and modern medical science is found in the condition known as trimethylaminuria, often described in literature and historical texts due to its distinctive symptom of creating a fish-like odor in those affected [16]. This metabolic disorder, where the body is unable to break down trimethylamine into trimethylamine oxide, results in the accumulation and release of this compound through sweat, urine, and breath, leading to significant social and psychological impacts on sufferers. The condition, referenced indirectly in historical observations and literary descriptions, echoes the ancient narrative's focus on bodily emanations and their effects. Shakespeare's reference to Caliban in The Tempest as having a fish-like smell might be seen as an intuitive grasp of medical reality, now fully understood in biochemical terms [17].

These stories and their modern interpretations provide a bridge between ancient wisdom and contemporary medical understanding, showing how mythological narratives can encode complex medical knowledge and insights. This integration of traditional knowledge with modern science not only enriches our understanding of health and disease but also underscores the potential of ancient texts to contribute to current medical practices and theories.

Symbolism and metaphors in mythological texts

In the rich tapestry of Indian mythology, health and disease are not merely physical states but are deeply intertwined with the moral, ethical, and spiritual fabric of the universe. The ancient texts and folklore of India, through their allegorical narratives, offer profound insights into the human condition, emphasizing the interconnectedness of the physical, mental, and spiritual realms.

Decoding Health Messages in Folktales

Indian folktales are replete with allegorical characters and events that convey intricate psychological themes, often mirroring the internal conflicts and healing processes faced by individuals. For instance, the story of "King Vikram and Betaal" serves as a metaphor for the ongoing dialogue within the human mind, reflecting the inner struggles and the quest for resolution many people grapple with. Similarly, "The Legend of Prince Dhruva" symbolizes the journey from emotional distress to inner peace, portraying a young boy's unwavering determination to attain self-realization and mental well-being.

These narratives employ symbolism to depict the complexities of mental health issues such as anxiety and depression. The tale of "King Shibi and the Hawk," for example, captures the emotional burden associated with depression, while "Raja Harischandra" explores themes of relentless honesty and the inner struggles brought on by anxiety. Furthermore, dissociative disorders, characterized by a disconnection from one's sense of self, find resonance in the story of "Shakuntala," which embodies themes of memory loss and regained identity.

Examples From Indian Epics

The Mahabharata, one of the great Indian epics, encompasses philosophical preaching, moral ways of living, and teachings of humanity, as exemplified by Lord Krishna's discourse to Arjuna in the Bhagavad Gita [18]. This epic also references the importance of Satsanga (the company of good, like-minded, and compatible people) and Sadachara (noble deeds) in preventing psychological disorders, highlighting the role of a wholesome diet in preventing somatic disorders. Such references directly align with Ayurvedic principles, emphasizing the holistic approach to health that encompasses both physical and mental well-being [19,20].

In the "Shantiparva" section of the Mahabharata, the psychosomatic disorders and their clinical picture are described, along with the influence of the Trigunas (Satva, Raja, and Tama) on the body and mind, in a manner similar to Ayurvedic teachings. This integration of philosophical and medical knowledge in the epic underscores the deep-rooted belief in the balance of physical elements (Vayu, Ushna, and Sheeta) and mental qualities (Sattva, Rajas, and Tamas) as the foundation of health [21].

The symbolism and metaphors present in Indian mythological texts and folktales not only enrich the cultural heritage but also offer timeless wisdom on health, disease, and healing. By decoding the health messages embedded in these narratives, one can gain insights into the holistic approach to well-being that characterizes ancient Indian medicine and philosophy. These stories, with their deep-rooted symbolism, continue to resonate with contemporary audiences, bridging the gap between ancient wisdom and modern medical understanding.

Ayurveda’s enduring legacy

Ayurveda, recognized as one of the oldest traditional systems of medicine globally, continues to influence modern medical practices and extend its reach internationally. Originating over 5,000 years ago, this holistic science has been integral to health and wellness, emphasizing balance and harmony between the mind, body, and spirit. Its principles, deeply rooted in the ancient schools of Hindu philosophical teachings, such as Vaisheshika and Nyaya, have guided diagnostic and treatment processes through logical and empirical approaches [22,23].

Ayurveda as a Separate Medical Practice

In India, Ayurveda is a household name and there will always be people who have knowledge about it. From the use of turmeric as an antiseptic to the use of papaya leaves for arthritis in chikungunya [24,25], Ayurveda is practiced by every household. Biases arise in the use of the pathy when patients think that Ayurveda can make survival possible in acute and decompensated stages and the clash with modern clinical medicine and allopathy becomes prominent there as the patient’s wishes and the doctor’s prescription no longer match [26,27]. The effect of Ayurveda in clinical medicine can be seen. The drug extraction process from different plant-based and fungi-based origins and certain synthetic molecules whose analogs are seen in the wild are just a few examples of how clinical medicine is influenced by Ayurveda and how Ayurveda can still show a path for the growth of clinical medicine [28-31].

Influence on Modern Medical Practices

The holistic approach of Ayurveda, focusing on curing diseases by restoring balance within the body and strengthening the host to prevent relapses, has been particularly influential in modern therapeutic practices. Ayurveda’s extensive pharmacopeia, which includes a wide array of herbs and minerals, forms the basis of many contemporary alternative medicine therapies. Its principles of personalized medicine recognizing the unique constitution of each individual are echoed in modern precision medicine, which tailors health care to individual genetic profiles, environments, and lifestyles [27,32].

The practice of Ayurvedic massage, known for alleviating vata and promoting overall well-being by reducing fatigue and stimulating the nervous system, has been adopted widely within integrative health practices globally. Additionally, the concept of Panchakarma, a detoxification therapy, underscores Ayurveda's influence on holistic cleansing practices embraced by wellness centers worldwide [33,34].

International Reach and Recognition

Ayurveda’s global impact is further evidenced by its integration into international health and wellness industries. The First International Congress on Ayurveda, held in Milan in 2009, marked a significant step in bridging Indian and Western medical philosophies, attracting over 400 participants and highlighting Ayurveda’s relevance to contemporary health discussions [35]. This event underscored the growing acceptance and integration of Ayurvedic principles in Western healthcare systems, promoting a holistic view of health that aligns with both traditional and modern scientific approaches.

Moreover, the World Health Organization (WHO) has recognized the importance of traditional and complementary medicine systems like Ayurveda, advocating for their integration into global health systems [36]. This includes setting standards and guidelines for the safe and effective use of herbal medicines, which are pivotal in Ayurvedic practice. The endorsement by global leaders, such as when the Prime Minister of India highlighted Ayurveda’s role in treating international dignitaries, further validates its efficacy and global appeal [36,37].

One of the most commonly used Ayurvedic techniques in the world is the application of turmeric as an antiseptic on the wound. Turmeric is also used in joint pain as a topical application. Many herbs and spices today are used for their health benefits, which are mentioned in Ayurveda.

In conclusion, Ayurveda’s enduring legacy is evident not only in its sustained practice within India but also in its expanding influence on global health paradigms. Its holistic approach, emphasis on balance and natural therapies, and integration with modern medical practices continue to make Ayurveda a significant contributor to global health care.

Integration of mythology in clinical practice

The profound connection between mythology and clinical practice extends beyond mere historical curiosity; it serves as a vital link in understanding and applying medical knowledge today. Myths, often dismissed as mere stories, actually carry deep symbolic meanings and possess a strong cultural appeal that can influence medical thinking and teaching [4,38]. These narratives become intertwined with clinical practices as they are shared routinely, gaining credence through repetition, until they comfortably settle into the gaps of medical education.

In the realm of medical education, myths can sometimes be mistaken for evidence, propagated through our tendency to simplify the complex or to generalize the specific [39]. This tendency can lead to the acceptance of intuitive but unsupported medical practices. However, myths also play a crucial role in informing the questions we ask and the research we engage in, shaping the methodological approaches in medicine. It is imperative to explore these connections critically, understanding both the benefits and the potential pitfalls of integrating mythological elements into clinical practice.

Lessons for Modern Medicine

Modern medicine can learn significantly from the integration of mythology into clinical practice. The narratives of myths provide a rich source of psychological and cultural insights that can enhance the therapeutic relationship between healthcare providers and patients. For instance, the mythopoetic approach helps bridge the personal experiences of patients, particularly those at life's crossroads or undergoing significant transitions, with universal themes found in mythology [4,40,41]. This connection can be especially powerful in contexts like depression or other mental health challenges, where patients may feel isolated in their experiences. Myths can offer comfort and a sense of belonging, suggesting that others have endured similar struggles and emerged transformed.

Mythology also enriches the medical practice by providing symbolic frameworks that can lead to deeper understanding and empathy. For example, the trials of Psyche in the myth of Eros and Psyche can be seen as a metaphor for personal growth through adversity, resonating with patients undergoing psychological or physical challenges [4,40]. This symbolic interpretation helps clinicians provide care that is not only technically proficient but also emotionally and spiritually supportive, fostering a holistic healing environment.

Ayurveda teaches us more than the mythology. It shows a way of life aligned with nature and how humans can benefit from the surrounding nature and how nature can benefit from humans. Yoga is a form of Ayurveda that is not only meant for treating diseases but for their prevention as well. Ayurveda teaches humans that living life is a long game and that you need to take care of your health every day to live not only longer but also a happier and fuller life.

Practical Applications Today

The practical applications of mythology in clinical practice are manifold. By incorporating mythological narratives and symbols into therapeutic settings, practitioners can address the often neglected spiritual or existential dimensions of healing. This approach can be particularly effective in palliative care, where understanding a patient’s spiritual and cultural background can play a crucial role in providing compassionate and personalized care [42,43].

Furthermore, the principles derived from mythological stories can inspire the development of therapeutic models that integrate the emotional, psychological, and spiritual dimensions of health. For example, the concept of the hero's journey, a common motif in many myths, can be adapted to help patients navigate the challenges of chronic illness or recovery from surgery, viewing their journey through a heroic lens that emphasizes resilience and transformation [1,3,44].

The integration of mythology in clinical practice not only enriches the therapeutic relationship but also encourages a more nuanced and compassionate approach to medicine. By embracing the lessons from ancient narratives, healthcare providers can offer care that is deeply resonant and culturally sensitive, ultimately leading to better patient outcomes and satisfaction.

## Conclusions

Ancient Indian mythology has significantly influenced clinical medicine, with its mythological underpinnings serving as the bedrock of holistic medical practices. These stories, not mere allegories, emphasize the parity between mind, body, and spirit, which is increasingly validated in modern therapeutic paradigms. The philosophy and methods detailed in the texts of Sushruta and Charaka, along with the allegorical tales of Dhanvantari and Bharadwaja, contribute significantly to the foundational principles of today's holistic medical approaches. The integration of mythology within clinical practice offers insights into the potential of ancient wisdom to inform and transform modern medicine. By embracing traditional narratives, healthcare practitioners gain access to a broader spectrum of understanding and empathy, fostering an environment where healing transcends the physical body to encompass the psychological and spiritual dimensions of well-being. This synthesis enriches the therapeutic landscape and offers a comprehensive framework for addressing the multidimensional aspects of health and disease.
